# Applicability of the five case model to African eHealth investment decisions

**DOI:** 10.1186/s12913-020-05526-6

**Published:** 2020-07-20

**Authors:** Sean C. Broomhead, Maurice Mars, Richard E. Scott, Tom Jones

**Affiliations:** 1 Department of TeleHealth, Nelson R. Mandela School of Medicine, University of KwaZulu–Natal, Durban, South Africa; 2 African Centre for eHealth Excellence, Cape Town, South Africa; 3 Health Information Systems Program, Waterkloof Ridge, Pretoria, South Africa; 4 NT Consulting – Global e-Health Inc., Calgary, Alberta Canada; 5 University of Calgary, Calgary, Alberta Canada

**Keywords:** eHealth, Digital health, Investment, Impact, Economics, Developing countries, Africa

## Abstract

**Background:**

eHealth programmes in African countries face fierce competition for scarce resources. Such initiatives should not proceed without adequate appraisal of their probable impacts, thereby acknowledging their opportunity costs and the need for appraisals to promote optimal use of available resources. However, since there is no broadly accepted eHealth impact appraisal framework available to provide guidance, and local expertise is limited, African health ministries have difficulty completing such appraisals. The Five Case Model, used in several countries outside Africa, has the potential to function as a decision-making tool in African eHealth environments and serve as a key component of an eHealth impact model for Africa.

**Methods:**

This study identifies internationally recognised metrics and readily accessible data sources to assess the applicability of the model’s five cases to African countries.

**Results:**

Ten metrics are identified that align with the Five Case Model’s five cases, including nine component metrics and one summary metric that aggregates the nine. The metrics cover the eHealth environment, human capital and governance, technology development, and finance and economics. Fifty-four African countries are scored for each metric. Visualisation of the metric scores using spider charts reveals profiles of the countries’ relative performance and provides an eHealth Investment Readiness Assessment Tool.

**Conclusion:**

The utility of these comparisons to strengthen eHealth investment planning suggests that the five cases are applicable to African countries’ eHealth investment decisions. The potential for the Five Case Model to have a role in an eHealth impact appraisal framework for Africa should be validated through field testing.

## Background

African countries experience high disease burdens compounded by resource shortages. This results in competition for available resources so that each decision to invest represents an opportunity cost that governments must weigh carefully to ensure optimal use of available funds [1]. eHealth initiatives offer potential to help improve health system performance and there is growing pressure to show positive impacts and health system benefits for each investment [2]. African governments are handicapped by a lack of evidence of which eHealth impact appraisal methodologies are applicable to support decision-making in African countries. This is further complicated by a diversity of views on what eHealth means [3], the role it should play [4], and benchmarks for good practice [5]. Without resolving these issues, the likelihood of optimal investment decisions being made diminishes.

A number of terms have been used to describe the use of Information Communication Technology (ICT) in the health sector, such as eHealth, digital health and health ICT, as well as sub-disciplines such as telemedicine and mobile health [6]. This paper uses eHealth and its World Health Organization (WHO) definition; “eHealth is the use of ICTs for health” [7]. While digital health frequently appears as an alternative to eHealth, there are recognised differences in its meaning [3].

Many eHealth initiatives fail [8, 9]. Therefore, eHealth should not attract public investment until its probable impacts have been appraised. The main reason for estimating impact is to ensure that the benefits realised from an investment justify the costs over time for key stakeholders, and rationalise the opportunity cost. This requires a value judgement tailored to local priorities such as access to services, Sustainable Development Goals (SDGs) and Universal Health Coverage (UHC) [10], and a way to balance the competing dimensions of value and affordability. An eHealth Impact model is a generic appraisal approach to support this type of decision-making [11]. For African countries, a methodology is needed to help those faced with making decisions about proposed eHealth initiatives to conduct prospective appraisals despite a scarcity of specialised economics, eHealth and other expertise [12].

There are numerous approaches to the assessment of economic impact in the health sector [13–15]. Some, such as the Health Impact Assessment approach [16, 17], extend beyond economic aspects to deal with broader societal impact, often referred to as socio-economic impact [18]. Few approaches are specific to eHealth, with notable exceptions such as the European eHealth IMPACT study [19], the Digital Health Impact Framework (DHIF) developed by the Asian Development Bank [20] and based on the Five Case Model, and a staged-based approach to integrating economic and financial evaluations specifically for mobile health initiatives [21].

The Five Case Model is described in The Green Book [22] and a User Manual [23]. Each case has a specific purpose, addressing the distinct questions summarised in Table 1. The Five Case Model is recommended by the United Kingdom [22, 24] and New Zealand [25] as a tool for promoting accountability for decisions about a variety of public spending initiatives, including eHealth. It provides an appraisal of the estimated probable value of an initiative’s options within the complex health system it operates in. This strengthens the justification for investing in initiatives that do well in the five cases, and justifies further research into its potential utility.

**Table 1:** Overview of the cases constituting the Five Case Model

	Strategic Case	Economic Case	Financial Case	Commercial Case	Management Case
**The primary question the case must answer about a proposed initiative**	Is it needed?	Is it value for money?	Is it affordable?	Is there a viable partnership model?	Is it achievable?
**Further questions each case should address**	Will the initiative further the country’s aims and objectives? Is there a clear case for change? Is there a theory of change?	Have economic cases been compared for a range of implementation options? Are the options meaningfully different (e.g., provide incremental change or new business models)? Have costs, benefits, sensitivity, optimism bias and risks been estimated? Have these parameters been adjusted for sensitivity, optimism bias and risk? Is it the best balance of cost, benefits and risk?	Are the costs realistic and affordable? Will the country be able to make the required funding available? Have options been considered for capital expenditure and leasing? Is the affordability sustainable?	Is there a supplier that can deliver any part of the initiative to be implemented? Can a value-for-money deal be secured with a supplier? How will suppliers be selected and assessed? Is there an appropriate Private Finance Initiative or Public Private Partnership option to be considered and evaluated?	Is the country capable of managing the envisaged initiative? Does the country have robust systems and processes in place?

The Five Case Model is a decision-making tool designed with the flexibility African countries need, such as balancing value against affordability constraints, and allowing progress despite limited human capacity to conduct complex eHealth economic appraisals. Nevertheless, the applicability of the five cases to African eHealth investments has not been assessed. In order to show this applicability, metrics are needed that are aligned to the five cases and relevant to African countries’ eHealth investment decisions. This study aims to identify appropriate metrics and data sources in order to judge the applicability of the Five Case Model in African eHealth settings, as an important step preceding field testing of the Five Case Model in Africa.

## Methods

To achieve the aim, readily accessible online data sources were explored to identify candidate metrics. The primary selection criteria were that each metric should provide information relevant to an eHealth issue aligned to one or more of the primary questions of the five cases, and be accessible online. Candidate metrics were then assessed for data availability from recognised sources such as the World Health Organization (WHO), International Telecommunications Union (ITU) and World Bank. Finally, the number of African countries for which the data were available was assessed. The initial intention was to select candidate metrics for which data were available for more than 80% of countries. However, the threshold was subsequently revised to 60% due to sparsity of data. Where less than 60% of African countries had available data, the metric was excluded. The remaining metrics became the component metrics of an eHealth Investment Readiness Assessment Tool.

Data for component metrics were collected for the fifty-four countries of the United Nations African region [26]. Where data were missing for a metric, values were set at zero to avoid recording progress that was not substantiated, except where more than a third of a metric’s data were missing, in which case the mean was used, since a zero value might reflect challenges with the metric’s data collection process rather than limited eHealth development. Thereafter, for each metric, values were reduced to a proportion of 1, where 1 was the maximum score possible and was assigned to the country with the highest score for that metric. An average of all component metrics provided a summary score that was used to rank overall country eHealth investment readiness. No weightings were applied. Scores were categorised as good (> 0.70), moderate (0.50–0.70) or poor (< 0.50).

Metric scores were analysed using spider charts, a graphical method of displaying multivariate data in a two-dimensional chart of three or more quantitative variables represented on axes starting from the same point. These visualisations were used to create multi-country profiles for five groups of countries: first, the countries with the highest five summary scores, and thereafter comparison of the countries with the highest, the lowest and the median scores for each of four regional economic communities of the African Union [27], the Arab Maghreb Union (AMU), East African Community (EAC), Economic Community of West African States (ECOWAS) and the Southern African Development Community (SADC).

## Results

Candidate metrics were identified based on the authors’ experience working in eHealth in African countries over many decades. This identified the following nineteen candidate metrics, arranged in four categories:

Category 1: eHealth development indicators

1. Global digital health index [28]
2. Global Observatory for eHealth (GOE) survey score [29]
3. Relative ranking among countries that have achieved 100% birth registration and 80% death registration
4. Relative ranking among countries that have conducted at least one population and housing census in the last 10 years
5. Service availability and readiness assessment [30]
6. Status of national eHealth strategy

Category 2: Financial and economic indicators

7. Current Health Expenditure (CHE) as a percentage of Gross Domestic Product (GDP) [31]
8. CHE per capita [32]
9. Government debt per capita
10. Proportion of total government spending on essential services
11. Rate of growth of real GDP in developing economies [33, 34]

Category 3: ICT development indicators

12. ICT Development Index (IDI) score [35, 36]
13. International Health Regulations (IHR) capacity and health emergency preparedness [37]
14. International Organization of Standardization ratings
15. Internet penetration score [38]
16. ITU percentage of individuals using the Internet [39]

Category 4: Workforce and governance indicators

17. Human Capital Index (HCI) score [40, 41]
18. Ibrahim Index of Governance in Africa score [42]
19. Rating agency risks assessment scores

Nine of the candidate metrics had sufficiently complete and readily accessible data. Table 2 lists the nine selected metrics and indicates which of the five cases each addresses and the percentage of countries for which data were found. For each metric, the most recent available data were used. The most recent GOE survey score was for 2015 and was the only source found for data about countries’ eHealth environments. In contrast, the most recent data for the status of eHealth Strategy were for 2018. The other seven data sources were for years from 2015 to 2017.

**Table 2:** Information about the selected metrics and the cases each metric is applicable to

Selected Metrics		Applicability of selected metrics to the Five Case Model’s five cases				
Type of indicator	Name of metric and year of data set used	Strategic	Economic	Financial	Commercial	Management
**eHealth development indicators**	1. Status of national eHealth strategy, 2018	X				
	2. GOE survey score, 2015	X	X	X	X	X
**Financial and economic indicators**	3. CHE as a percentage of GDP, 2015			X		
	4. CHE per capita, 2015			X		
	5. Rate of growth of real GDP, 2017		X	X		
**ICT development indicators**	6. IDI score, 2017	X				X
	7. Internet penetration score, 2016/2017	X			X	X
**Workforce and governance indicators**	8. HCI score, 2017		X	X	X	X
	9. Ibrahim Governance Index score, 2016	X	X	X	X	X

Ten candidate metrics were rejected for the following reasons:

- Data sets incomplete, in the case of the digital health readiness index
- Data sets not readily accessible, in the case of government debt per capita and proportion of total government spending on essential services
- No easily interpretable summary score, in the case of the service availability and readiness assessment, rating agency assessments, IHR capacity and health emergency preparedness, and membership of the IOS
- Similar, more appropriate metrics identified, as in the case of relative ranking among countries that have conducted at least one population and housing census in the last 10 years, and the relative ranking among countries that have achieved 100% birth registration and 80% death registration.

Descriptions of each of the selected metrics and the approaches to missing data are provided in Table 3.

**Table 3:** Descriptions of selected metrics and approach to missing data

Metric	Description	Percentage of countries with available data	Handling of missing data
1. Status of national eHealth strategy	A score of the extent to which a country has addressed each of the following four aspects pertinent to eHealth in a published document: UHC strategy or policy, eHealth policy or strategy, health information systems policy or strategy, national telehealth policy or strategy. Information was derived from the eHealth Strategy score in the WHO GOE Survey, updated for countries that had published more recent strategy documents found on Internet search	74%	Data values were set to zero for fourteen countries where data were not available
2. GOE survey score	An aggregate percentage of the maximum points a country can score for all key aspects evaluated in the survey	61%	For 21 countries whose data were not available the mean was applied
3. CHE as a percentage of GDP	The level of current health expenditure expressed as a percentage of GDP. Estimates of current health expenditures include healthcare goods and services consumed during each year. This indicator does not include capital health expenditures such as buildings, machinery, IT and stocks of vaccines for emergency or outbreaks [31].	96%	Data values were set to zero for two countries where data were not available
4. CHE per capita	The level of current expenditures on health per capita in current US dollars. Estimates of current health expenditures include healthcare goods and services consumed during each year [32].	96%	Data values were set to zero for two countries where data were not available
5. Rate of growth of real GDP	A measure of the annual rate of change of a nation’s gross domestic product (GDP) after adjusting for the effect that inflation or deflation has on the economy [33, 34].	96%	Data values were set to zero for two countries where data were not available
6. IDI score	A composite index that combines fourteen indicators on ICT access, use and skills, capturing key aspects of ICT development in one measure that allows for comparisons to be made between countries and over time of the level of ICT development across the world [35]. It is published by the ITU based on internationally agreed ICT indicators.	87%	Data values were set to zero for seven countries where data were not available
7. Internet penetration score	An average of two data elements: Internet penetration percentage [38] calculated by dividing Internet user estimates by published population estimates and the IDI score for the percentage of individuals using the Internet [39].	100%	Data set complete
8. HCI score	Quantifies the contribution of health and education to the productivity of the next generation of workers [40]. The HCI index score ranges from zero to one and measures the productivity of a child born today as a future worker relative to the benchmark of full health and complete education [41].	83%	Data values were set to zero for nine countries where data were not available
9. Ibrahim Governance Index score	A tool that measures and monitors governance performance in African countries [42]. Performance is measured across four key components: safety and rule of law, participation and human rights, sustainable economic opportunity, and human development. A summary score provides an indication of overall governance performance.	100%	Data set complete

**Fig. 1:**
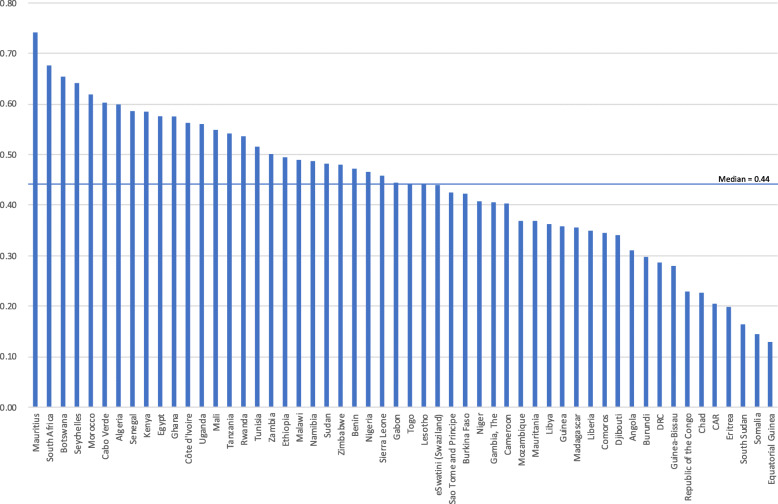
Ranking of African country eHealth investment readiness using summary metric scores

Figure 1 shows the country scores and relative rankings of the summary metric. Detailed scores are provided in Tables 4 and 5.

**Table 4:** Metric scores

	Summary metric	1. Strategy	2. GOE survey	3. CHE as a % of GDP	4. CHE per capita	5. Growth of real GDP	6. IDI	7. Internet penetration	8. HCI	9. Ibrahim Governance Index
Mean	0.44	0.40	0.54	0.32	0.23	0.49	0.42	0.41	0.50	0.62
Mean + one standard deviation	0.58	0.74	0.73	0.49	0.48	0.86	0.67	0.68	0.75	0.79
Mean + two standard deviations	0.72	1.08	0.93	0.65	0.74	1.22	0.93	0.94	0.99	0.95
1. Mauritius	0.74	0.50	0.54	0.30	1.00	0.50	1.00	0.91	0.92	1.00
2. South Africa	0.68	1.00	0.47	0.45	0.93	0.09	0.84	0.85	0.60	0.86
3. Botswana	0.65	0.50	0.80	0.33	0.77	0.57	0.78	0.62	0.63	0.89
4. Seychelles	0.64	0.25	0.61	0.19	0.97	0.00	0.86	1.00	1.00	0.90
5. Morocco	0.62	0.50	0.62	0.30	0.32	0.60	0.81	0.95	0.74	0.74
6. Cabo Verde	0.60	1.00	1.00	0.26	0.29	0.56	0.84	0.59	0.00	0.89
7. Algeria	0.60	0.50	0.62	0.39	0.58	0.40	0.79	0.69	0.77	0.66
8. Senegal	0.59	0.75	0.82	0.22	0.07	0.91	0.45	0.67	0.62	0.76
9. Kenya	0.58	0.75	0.56	0.28	0.14	0.74	0.49	0.80	0.76	0.73
10. Egypt	0.58	0.75	0.54	0.23	0.31	0.53	0.79	0.71	0.72	0.61
11. Ghana	0.58	0.25	0.80	0.32	0.16	0.97	0.69	0.54	0.65	0.80
12. Côte d’Ivoire	0.56	0.75	0.62	0.30	0.15	1.00	0.53	0.53	0.52	0.67
13. Uganda	0.56	0.75	0.92	0.40	0.09	0.74	0.37	0.51	0.56	0.69
14. Mali	0.55	1.00	0.71	0.32	0.08	0.76	0.37	0.60	0.47	0.64
15. Tanzania	0.54	1.00	0.54	0.33	0.06	0.93	0.31	0.41	0.59	0.71
16. Rwanda	0.54	0.75	0.56	0.43	0.11	0.87	0.37	0.39	0.55	0.79
17. Tunisia	0.52	0.00	0.07	0.37	0.51	0.40	0.82	0.92	0.75	0.80
18. Zambia	0.50	0.75	0.62	0.30	0.14	0.46	0.43	0.53	0.58	0.71
19. Ethiopia	0.49	0.75	0.83	0.22	0.05	0.93	0.28	0.24	0.57	0.59
20. Malawi	0.49	0.75	0.78	0.51	0.07	0.54	0.30	0.17	0.60	0.70
21. Namibia	0.49	0.00	0.54	0.49	0.84	−0.14	0.66	0.49	0.64	0.87
22. Sudan	0.48	0.75	0.51	0.34	0.30	0.60	0.43	0.44	0.56	0.40
23. Zimbabwe	0.48	0.50	0.87	0.56	0.19	0.00	0.50	0.50	0.65	0.56
24. Benin	0.47	0.75	0.44	0.22	0.06	0.77	0.33	0.36	0.60	0.72
25. Nigeria	0.47	1.00	0.54	0.20	0.19	0.13	0.44	0.60	0.50	0.59
26. Sierra Leone	0.46	0.25	0.54	1.00	0.21	0.79	0.00	0.18	0.52	0.64
27. Gabon	0.44	0.00	0.54	0.15	0.39	0.16	0.70	0.75	0.67	0.64
28. Togo	0.44	0.50	0.54	0.36	0.07	0.71	0.37	0.18	0.61	0.64
29. Lesotho	0.44	0.50	0.20	0.46	0.18	0.41	0.52	0.43	0.55	0.71
30. eSwatini (Swaziland)	0.44	0.75	0.54	0.38	0.46	0.14	0.00	0.48	0.60	0.60
31. Sao Tome and Principe	0.42	0.00	0.54	0.54	0.32	0.71	0.53	0.44	0.00	0.75
32. Burkina Faso	0.42	0.25	0.48	0.30	0.07	0.91	0.32	0.26	0.54	0.66
33. Niger	0.41	0.50	0.83	0.39	0.05	0.74	0.00	0.07	0.47	0.62
34. Gambia, The	0.41	0.50	0.43	0.37	0.06	0.37	0.44	0.29	0.59	0.60
35. Cameroon	0.40	0.00	0.54	0.28	0.13	0.54	0.40	0.58	0.58	0.58
36. Mozambique	0.37	0.00	0.54	0.30	0.06	0.59	0.39	0.27	0.53	0.64
37. Mauritania	0.37	0.25	0.43	0.25	0.11	0.54	0.38	0.28	0.52	0.55
38. Libya	0.36	0.00	0.54	0.00	0.00	1.00	0.70	0.62	0.00	0.41
39. Guinea	0.36	0.00	0.54	0.25	0.05	0.80	0.30	0.17	0.55	0.56
40. Madagascar	0.36	0.25	0.47	0.28	0.04	0.61	0.29	0.09	0.55	0.61
41. Liberia	0.35	0.00	0.54	0.83	0.14	0.41	0.00	0.12	0.47	0.63
42. Comoros	0.35	0.25	0.19	0.44	0.12	0.40	0.31	0.19	0.60	0.61
43. Djibouti	0.34	0.00	0.54	0.24	0.16	0.97	0.34	0.25	0.00	0.57
44. Angola	0.31	0.00	0.54	0.16	0.21	0.27	0.33	0.25	0.53	0.48
45. Burundi	0.30	0.25	0.55	0.45	0.05	0.00	0.25	0.08	0.56	0.49
46. DRC	0.29	0.00	0.54	0.23	0.04	0.43	0.26	0.10	0.54	0.43
47. Guinea-Bissau	0.28	0.25	0.20	0.38	0.08	0.77	0.25	0.08	0.00	0.51
48. Republic of the Congo	0.23	0.00	0.54	0.19	0.12	−0.09	0.00	0.16	0.62	0.53
49. Chad	0.23	0.00	0.54	0.25	0.07	0.01	0.22	0.08	0.43	0.43
50. CAR	0.20	0.00	0.25	0.26	0.03	0.67	0.18	0.07	0.00	0.37
51. Eritrea	0.20	0.00	0.54	0.18	0.06	0.46	0.16	0.02	0.00	0.36
52. South Sudan	0.16	0.25	0.16	0.14	0.06	0.00	0.00	0.19	0.45	0.25
53. Somalia	0.14	0.50	0.22	0.00	0.00	0.36	0.00	0.08	0.00	0.14
54. Equatorial Guinea	0.13	0.00	0.17	0.15	0.55	−0.84	0.32	0.37	0.00	0.45

**Table 5:** Matrix of correlations between individual metrics

Correlations matrix	Summary metric	eHealth strategy	GOE survey	CHE as a % of GDP	CHE per capita	Growth of real GDP	IDI	Internet penetration	HCI	Ibrahim Governance Index
Summary metric	1.00	0.61	0.53	0.23	0.52	0.32	0.73	0.78	0.60	0.85
Correlation with eHealth Strategy	0.61	1.00	0.39	0.05	0.05	0.24	0.19	0.34	0.25	0.33
Correlation with GOE survey	0.53	0.39	1.00	0.12	0.00	0.30	0.20	0.19	0.21	0.39
Correlation with CHE as a % GDP	0.23	0.05	0.12	1.00	0.10	0.07	−0.13	−0.10	0.19	0.34
Correlation with CHE per capita	0.52	0.05	0.00	0.10	1.00	−0.42	0.65	0.65	0.36	0.59
Correlation with Growth of real GDP	0.32	0.24	0.30	0.07	−0.42	1.00	0.03	−0.03	−0.07	0.17
Correlation with IDI	0.73	0.19	0.20	−0.13	0.65	0.03	1.00	0.84	0.35	0.66
Correlation with internet penetration	0.78	0.34	0.19	−0.10	0.65	− 0.03	0.84	1.00	0.47	0.64
Correlation with HCI	0.60	0.25	0.21	0.19	0.36	−0.07	0.35	0.47	1.00	0.52
Correlation with Ibrahim Governance Index	0.85	0.33	0.39	0.34	0.59	0.17	0.66	0.64	0.52	1.00

Mauritius achieved the highest summary metric score and was in the top five for five other metrics: CHE per capita (1st), IDI (1st), Ibrahim Governance Index (1st), HCI (2nd) and Internet penetration (4th). For the other four metrics Mauritius did not score in the top 20 countries.

Seven metrics showed greater than 0.50 correlation with the summary metric, namely the Ibrahim Governance Index (0.85), Internet penetration (0.78), ICT Development Index (IDI) (0.73), eHealth Strategy (0.61), Human Capital Index (HCI) (0.60), Global Observatory for eHealth (GOE) survey (0.53) and Current Health Expenditure (CHE) per capita (0.52). Correlation between component metrics was generally low, except between IDI and Internet penetration (0.84). Other correlations greater than 0.50 were between the Ibrahim Governance index and four other metrics: IDI (0.66), Internet penetration (0.64), CHE per capita (0.59), and HCI (0.52), and between CHE per capita and the two ICT metrics, Internet penetration (0.65) and IDI (0.65).

**Fig. 2:**
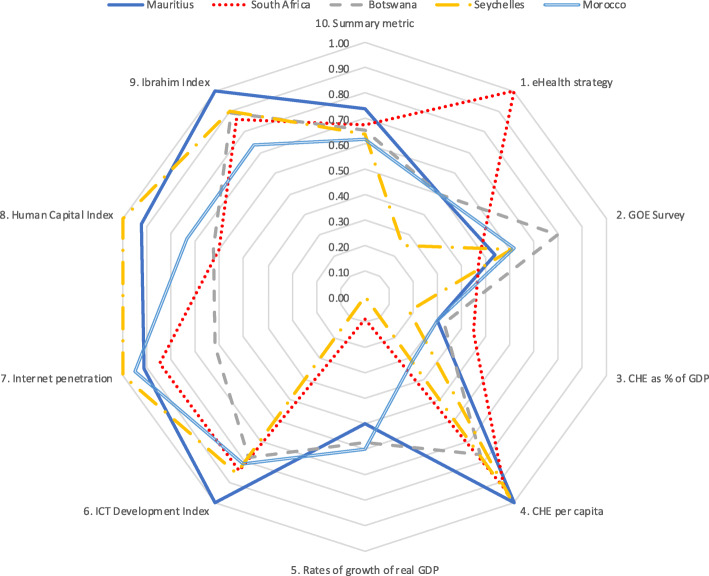
Spider chart of countries with highest five summary metric scores

**Fig. 3:**
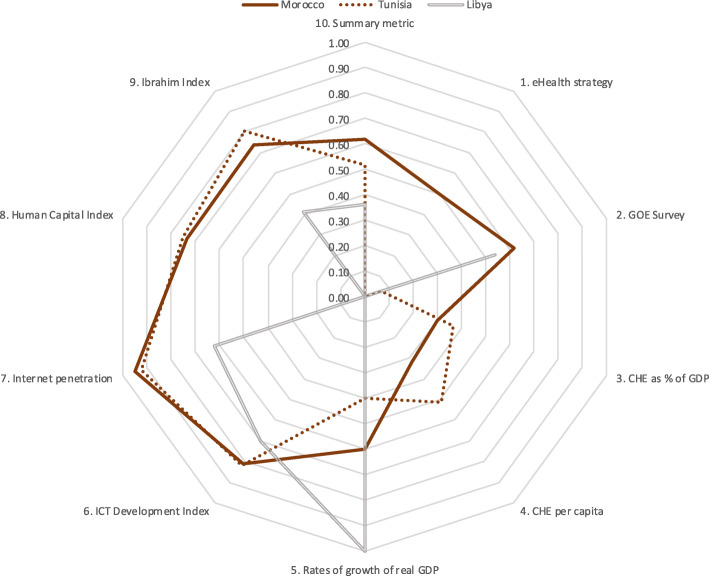
Spider chart comparison of three countries from the AMU: Morocco, Tunisia and Libya

**Fig. 4:**
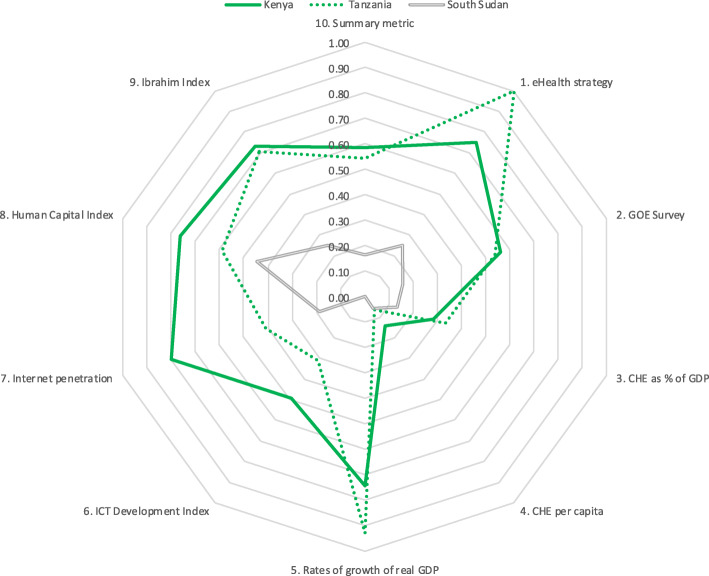
Spider chart comparison of three EAC countries: Kenya, Tanzania and South Sudan

**Fig. 5:**
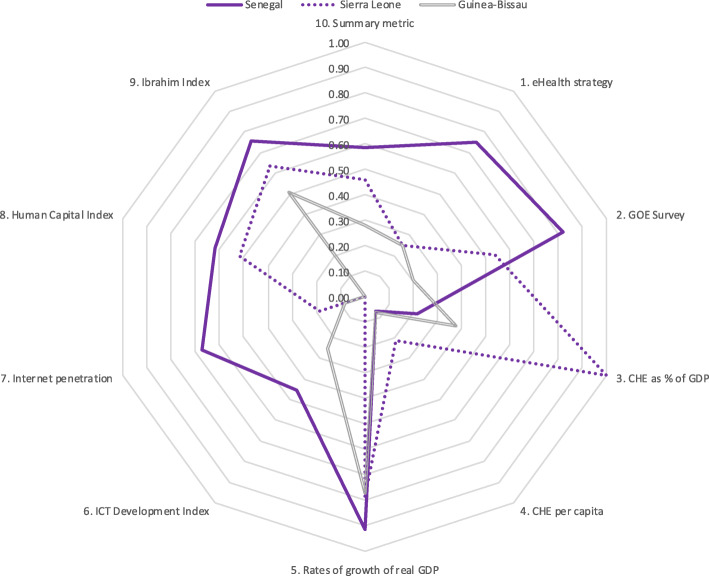
Spider chart comparison of three ECOWAS countries: Senegal, Sierra Leone and Guinea-Bissau

**Fig. 6:**
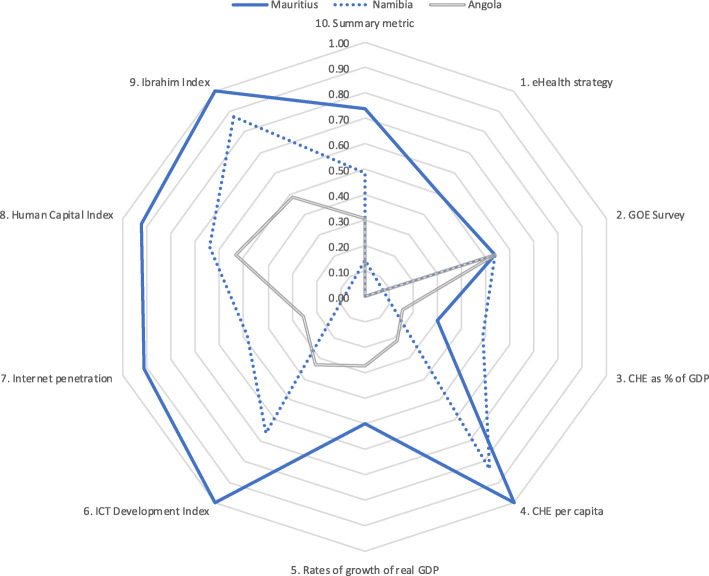
Spider chart comparison of three SADC countries: Mauritius, Namibia and Angola

Results for five country groupings are provided in Tables 6, 7, 8, 9, 10. Table 6 is of five countries that scored highest on the summary metric. Tables 7, 8, 9, 10 each show the scores for three countries (highest scoring, lowest scoring and the median) from each of four economic regions. To aid comparison, these data are presented in Tables 6, 7, 8, 9, 10 and the corresponding spider charts in Figs. 2, 3, 4, 5, 6 where the axes represent metrics and are arranged radially (1–10), with the scores for each metric plotted and linked.

**Table 6:** Metric scores for countries with top five summary metric scores Mauritius, South Africa, Botswana, Seychelles, Morocco

	Summary metric	1. Strategy	2. GOE Survey	3. CHE as a % of GDP	4. CHE per capita	5. Growth of real GDP	6. IDI	7. Internet penetration	8. HCI	9. Ibrahim Governance
Mauritius	**0.74**	0.50	0.54	*0.30*	**1.00**	0.50	**1.00**	**0.91**	**0.92**	**1.00**
South Africa	0.68	**1.00**	0.47	*0.45*	**0.93**	*0.09*	**0.84**	**0.85**	0.60	**0.86**
Botswana	0.65	0.50	**0.80**	*0.33*	**0.77**	0.57	**0.78**	0.62	0.63	**0.89**
Seychelles	0.64	0.25	0.61	*0.19*	**0.97**	*0.00*	**0.86**	**1.00**	**1.00**	**0.90**
Morocco	0.62	0.50	0.62	*0.30*	*0.32*	0.60	**0.81**	**0.95**	**0.74**	**0.74**

Legend: > **0.70 (Good)**, 0.50–0.70 (Moderate), *< 0.50 (Poor)*

**Table 7:** Metric scores for selected North African countries Morocco, Tunisia and Libya

	Summary metric	1. Strategy	2. GOE Survey	3. CHE as a % of GDP	4. CHE per capita	5. Growth of real GDP	6. IDI	7. Internet penetration	8. HCI	9. Ibrahim Governance
Morocco	0.62	0.50	0.62	*0.30*	*0.32*	0.60	**0.81**	**0.95**	**0.74**	**0.74**
Tunisia	0.52	*0.00*	*0.07*	*0.37*	0.51	*0.40*	**0.82**	**0.92**	**0.75**	**0.80**
Libya	*0.36*	*0.00*	0.54	*0.00*	*0.00*	**1.00**	0.70	0.62	*0.00*	*0.41*

Legend: **> 0.70 (Good),** 0.50–0.70 (Moderate), *< 0.50 (Poor)*

**Table 8:** Metric scores for selected EAC countries Kenya, Tanzania, South Sudan

	Summary metric	1. Strategy	2. GOE Survey	3. CHE as a % of GDP	4. CHE per capita	5. Growth of real GDP	6. IDI	7. Internet penetration	8. HCI	9. Ibrahim Governance
Kenya	0.58	**0.75**	0.56	*0.28*	*0.14*	**0.74**	*0.49*	**0.80**	**0.76**	**0.73**
Tanzania	0.54	**1.00**	0.54	*0.33*	*0.06*	**0.93**	*0.31*	*0.41*	0.59	**0.71**
South Sudan	*0.16*	*0.25*	*0.16*	*0.14*	*0.06*	*0.00*	*0.00*	*0.19*	*0.45*	*0.25*

Legend: **> 0.70 (Good),** 0.50–0.70 (Moderate), *< 0.50 (Poor)*

**Table 9:** Metric scores for selected ECOWAS countries Senegal, Sierra Leone, Guinea-Bissau

	Summary metric	1. Strategy	2. GOE Survey	3. CHE as a % of GDP	4. CHE per capita	5. Growth of real GDP	6. IDI	7. Internet penetration	8. HCI	9. Ibrahim Governance
Senegal	0.59	**0.75**	**0.82**	*0.22*	*0.07*	**0.91**	*0.45*	0.67	0.62	**0.76**
Sierra Leone	*0.46*	*0.25*	0.54	**1.00**	*0.21*	**0.79**	*0.00*	*0.18*	0.52	0.64
Guinea-Bissau	*0.28*	*0.25*	*0.20*	*0.38*	*0.08*	**0.77**	*0.25*	*0.08*	*0.00*	0.51

Legend: **> 0.70 (Good)**, 0.50–0.70 (Moderate), *< 0.50 (Poor)*

**Table 10:** Metric scores for selected SADC countries Mauritius, Namibia, Angola

	Summary metric	1. Strategy	2. GOE Survey	3. CHE as a % of GDP	4. CHE per capita	5. Growth of real GDP	6. IDI	7. Internet penetration	8. HCI	9. Ibrahim Governance
Mauritius	**0.74**	0.50	0.54	*0.30*	**1.00**	0.50	**1.00**	**0.91**	**0.92**	**1.00**
Namibia	*0.49*	*0.00*	0.54	*0.49*	**0.84**	*(0.14)*	0.66	*0.49*	0.64	**0.87**
Angola	*0.31*	*0.00*	0.54	*0.16*	*0.21*	*0.27*	*0.33*	*0.25*	0.53	0.48

Legend: **> 0.70 (Good),** 0.50–0.70 (Moderate), *< 0.50 (Poor)*

In the highest scoring group there are results from Mauritius (0.74), South Africa (0.68), Botswana (0.65), Seychelles (0.64) and Morocco (0.62). All five countries scored above 0.70 on the Ibrahim Governance Index (> 0.73) and IDI (> 0.77). Internet penetration scores were good (> 0.84) for all except Botswana (0.62). Despite these high scores, all five countries had poor scores for CHE as a % of GDP and moderate or poor for real GDP growth rates. The results are shown in Table 6 and Fig. 2.

Three of the five AMU member countries were compared. Morocco scored highest (0.62), Libya lowest (0.36) and Tunisia was the median (0.52). Libya achieved the highest score for growth of real GDP out of all 54 countries and Morocco and Tunisia achieved good scores for IDI, Internet penetration, HCI and governance. All other scores were moderate or poor. The results are in Table 7 and Fig. 3.

Three of the six EAC member countries were compared. Kenya scored highest (0.58), South Sudan lowest (0.16) and Tanzania was the median (0.54). Kenya and Tanzania scored above 0.70 for strategy, growth of real GDP and governance. Kenya also scored above 0.70 for Internet penetration and HCI. All three countries’ scores were poor for CHE per capita and CHE as a percentage of GDP. South Sudan’s scores were poor for all metrics. The results are in Table 8 and Fig. 4.

Three of the fifteen ECOWAS member states were compared. Senegal scored highest (0.59), Guinea-Bissau lowest (0.28) and Sierra Leone was the median (0.46). All three countries achieved good scores for growth of real GDP and poor scores for CHE per capita and IDI. Senegal’s scores were good for strategy, GOE survey and governance and Sierra Leone scored the highest out of all 54 countries for CHE as a percentage of GDP. All other scores were moderate or poor. The results are in Table 9 and Fig. 5.

Finally, three member states of SADC were compared. Mauritius scored highest (0.74), Angola lowest (0.31) and Namibia was the median (0.49). As discussed earlier, Mauritius was the only country to score above 0.70 for the summary metric. Mauritius surpassed Namibia and Angola in all metrics except the GOE survey, where Mauritius, Namibia and Angola scored the same (0.54), and CHE as a % GDP, where Namibia scored higher (0.49) than Mauritius (0.30). Namibia and Angola had poor scores for eHealth strategy, CHE as a % GDP, growth of real GDP and Internet penetration. The results are in Table 10 and Fig. 6.

## Discussion

Analysis of a country’s relative ranking on each component metric, and the summary metric, can be used to identify aspects where further development would contribute to eHealth investment strengthening. The summary metric provides an overall indication of a country’s eHealth investment readiness, relative to other countries. The inconsistency of data source years is a limitation, since a country’s economic condition, ICT development and eHealth development may vary from year to year. Future publication of an updated tool using metrics from a single, recent year – should they become available – would be of value.

**Fig. 7:**
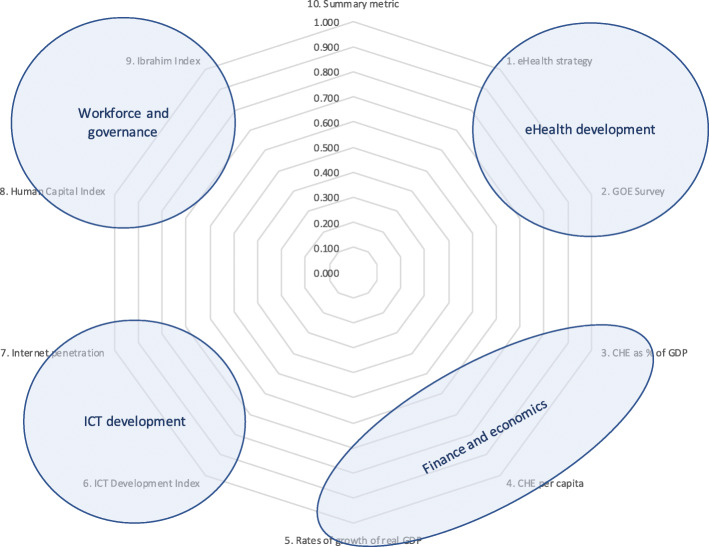
Issues represented by each of the spider chart quadrants

Comparison of the component metric profiles of regional country groupings can help those countries identify good practices to be shared with neighbouring countries. Individual metrics can hide nuances, therefore exploring all metrics for each country under evaluation is encouraged. Similarly, comparing countries’ profiles provides additional insights illustrated by the varying patterns seen on the spider charts. Scoring less than 1.00 for a metric shows underperformance against peers, and represents an opportunity for improvement. Comparison of the scoring patterns can reveal individual and/or regional performance in each of these quadrants: bottom and right lower quadrant for financial and economic indicators, left lower quadrant for two ICT development indicators, left upper quadrant for human capital and governance, and right upper quadrant for development of the eHealth environment (Fig. 7).

Using the study findings, each African country can review its metric scores, plot its spider chart to show its performance, and use the results to establish an eHealth investment strengthening plan. For example, despite having the highest summary score, Mauritius’ results identified four areas that, if strengthened, will improve the likelihood of successful eHealth investment. These included updating its eHealth strategy, addressing aspects of the GOE survey that scored poorly, growing the Mauritian economy and lobbying for more allocation of the fiscus to health.

Countries with an eHealth Strategy, relatively high GDP and health spend per capita, and high governance scores, such as Mauritius, South Africa and Botswana, can apply the Five Case Model to improve eHealth investment decisions. Countries with an eHealth Strategy and high governance score, but low CHE scores, such as Kenya, Morocco and Senegal, should start by focusing on the economics and finance aspects of their eHealth programmes. Countries with an eHealth Strategy and low governance score, such as Nigeria, should focus on governance strengthening, as a foundational requirement for eHealth investment.

Regional spider charts help to illustrate this analysis. Thus, data for the AMU (Fig. 3) suggest that while Morocco and Tunisia show similar patterns, Tunisia remains hampered by lack of an eHealth Strategy retarding its eHealth development. Changing this will remain challenging while growth of real GDP and CHE metrics remain low, represented on the spider chart as low scores on the right, lower quadrant. Despite Libya’s generally lower than average performance, Libya scores well on growth of real GDP (2nd), far higher than Morocco (22nd) and Tunisia (39th). This, combined with a moderate IDI score (0.70), sets the stage for Libya to craft an eHealth Strategy to guide the beginning of eHealth investments.

In the EAC (Fig. 4), Kenya and Tanzania have similar summary metric scores and high scores for strategy, growth rates of real GDP and governance, yet important differences, such as Kenya’s higher score on Internet penetration. If the region considered identifying a country lead for key elements, it would include Kenya leading on connectivity. South Sudan scores are poor on all metrics, though with a slight shift to the left caused by the HCI score (0.45), which could indicate potential warranting further development. A dominant feature of the EAC spider chart is poor scores on the two CHE metrics, represented by the “missing” bottom right quadrant, highlighting the need for growth to include more fiscal allocations to health.

In the ECOWAS (Fig. 5), all three countries show good growth of real GDP, though CHE per capita remains poor and IDI is poor. Each of the spider chart quadrants has some activity, which may indicate that a collaborative regional approach will prove fruitful. Sierra Leone has achieved the highest score on CHE as a percentage of GDP (1st), though has inadequate eHealth Strategy (36th) and poor GOE survey scores (37th). An opportunity could be to develop a new eHealth Strategy, fuelled by CHE priorities. Promising governance rankings in Senegal (10th) underpin the growth of real GDP and a regional eHealth leadership role for Senegal.

The SADC spider chart (Fig. 6), shows a marked “lean” towards the left caused by low scores in the two eHealth implementation metrics in the top right quadrant. Namibia’s poor eHealth strategy score may help to explain why, despite promising rankings on governance (5th) and IDI (13th), the GOE survey score remains low (33rd). Angola is constrained by poor scores on strategy, CHE metrics, IDI and governance. A regional strategy that includes collaboration to share good practices, particularly to improve SADC country’s eHealth strategies, might prove useful.

Correlation analysis provides information about relationships between component metrics. Correlations above 0.75 between the summary metric and two component metrics, the Ibrahim Governance Index (0.85) and Internet penetration (0.78), suggest that either of these would provide a reasonable surrogate indicator of overall eHealth investment readiness. Correlation between component metrics shows modest correlation for Ibrahim Governance Index and IDI (0.66), and Ibrahim Governance Index and Internet penetration (0.64). These are consistent with suggestions that ICT development plays a role in promoting good governance [43, 44] and may suggest that governance is a requirement for countries to make productive eHealth investments. Correlation between health expenditure per capita and ICT development (0.65) underpins the importance of addressing affordability issues and may support suggestions that ICT initiatives themselves contribute positively to economic growth [45].

The metrics used to develop the eHealth Investment Readiness Assessment Tool reflect aspects of eHealth investment that are aligned to the five cases. The tool highlights countries’ strengths and weaknesses, thereby providing information for targeted eHealth investment plans. It also helps to identify strengths in neighbouring countries to support collaborative partnerships for regional eHealth investment. This demonstrates the applicability of the Five Case Model to African eHealth investment decisions. The Five Case Model should now be validated through in-country field testing, by designing a tool based on the five cases and testing its utility to help decision makers select an appropriate initiative for investment from among promising candidates.

## Conclusion

The absence of recognised eHealth impact appraisal frameworks in regular use in African countries increases the opportunity cost of eHealth and the risk that investments will not produce optimal net benefits. The eHealth Investment Readiness Assessment Tool presented in this study ranked fifty-four African countries and profiled potential approaches for country and regional eHealth investment strengthening plans using metrics relevant to eHealth and aligned to the Five Case Model. The results illustrate the applicability of the Five Case Model for African eHealth investment decisions to serve as a component of an eHealth impact model for Africa. Whilst this study used African countries as the exemplar, the approach is likely to be useful elsewhere, particularly in Low and Middle Income Countries (LMICs), and complements recent developments such as the DHIF. Further scrutiny of the approach and assessment of its eHealth investment strengthening utility is encouraged.

## Data Availability

The datasets utilised for this study are available from the corresponding author on reasonable request, or from the following public repositories: • Global diffusion of eHealth: Making universal health coverage achievable. Report of the third global survey on eHealth. Geneva: Global Observatory for eHealth, World Health Organization; 2016. http://africahealthforum.afro.who.int/first-edition/IMG/pdf/global_diffusion_of_ehealth_-_making_universal_health_coverage_achievable.pdf. Accessed 09 Mar 2019. • Current health expenditure (CHE) as percentage of gross domestic product World Health Organization. http://apps.who.int/gho/data/view.main.GHEDCHEGDPSHA2011v. Accessed 04 Feb 2019. • Current health expenditure per capita. The World Bank; 2015. https://data.worldbank.org/indicator/SH.XPD.CHEX.PC.CD. Accessed 04 Feb 2019. • World Economic Situation and Prospects. United Nations, 2018. https://www.un.org/development/desa/dpad/wp-content/uploads/sites/45/publication/WESP2018_Full_Web-1.pdf. Accessed 04 Feb 2019.ITU Development Index. ITU; 2017. https://www.itu.int/net4/ITU-D/idi/2017/index.html. Accessed 09 Mar 2019. • Measuring the Information Society Report Geneva: International Telecommunications Union, 2017. https://www.itu.int/en/ITU-D/Statistics/Documents/publications/misr2017/MISR2017_Volume1.pdf. Accessed 04 Mar 2019. • Internet Users Statistics for Africa. 2017. https://www.internetworldstats.com/stats1.htm. Accessed 04 Feb 2019. • Percentage of Individuals using the Internet. International Telecommunications Union; 2016. https://www.itu.int/en/ITU-D/Statistics/Pages/stat/default.aspx. Accessed 27 Feb 2019. • Human Capital Index. World Bank; 2017. http://www.worldbank.org/en/publication/human-capital#Data. Accessed 09 Mar 2019. • Ibrahim Index of Governance in Africa 2017. http://iiag.online/. Accessed 04 Feb 2019.

## Abbreviations

AMU: Arab Maghreb Union
CHE: Current Health Expenditure
DHIF: Digital Health Impact Framework
EAC: East African Community
ECOWAS: Economic Community of West African States
GDP: Gross Domestic Product
GOE: Global Observatory of eHealth
HCI: Human Capital Index
ICT: Information Communication Technology
IDI: ICT Development Index
IHR: International Health Regulations
ITU: International Telecommunication Union
LMICs: Low and Middle Income Countries
PFI: Private Finance Initiative
PPP: Public Private Partnership
SADC: South African Development Community
SDGs: Sustainable Development Goals
UHC: Universal Health Coverage
WHO: World Health Organization
